# Network pharmacology and experimental study of phenolic acids in salvia miltiorrhiza bung in preventing ischemic stroke

**DOI:** 10.3389/fphar.2023.1108518

**Published:** 2023-01-27

**Authors:** Chengdi Liu, Lida Du, Sen Zhang, Haigang Wang, Linglei Kong, Guanhua Du

**Affiliations:** ^1^ Department of Pharmacy, Affiliated Beijing Friendship Hospital, Capital Medical University, Beijing, China; ^2^ Beijing Key Laboratory of Drug Targets Identification and Drug Screening, Institute of Materia Medica, Chinese Academy of Medical Sciences and Peking Union Medical College, Beijing, China; ^3^ Department of Surgery, University of Toronto, Toronto, ON, Canada

**Keywords:** phenolic acids, salvianolic acid A, ischemic stroke, network pharmacology, prevention drug, antiplatelet experiment

## Abstract

At present, the preventive effect of ischemic stroke is not ideal, and the preventive drugs are limited. Danshen, the dried root of Salvia miltiorrhiza Bge, is a common medicinal herb in Traditional Chinese Medicine, which has been used for the treatment of cardiovascular diseases for many years. Phenolic Acids extracted from danshen, which showed multiple biological activities, have been developed as an injection for the treatment of ischemic stroke. However, its preventive effect on ischemic stroke has not been fully reported. The current study aimed to identify the potential active phenolic acids for the prevention of ischemic stroke and explore its mechanism using network pharmacology and experimental analyses. The targets of phenolic acids and ischemic stroke were obtained from public databases. Network pharmacology predicted that 35 kinds of phenolic acids had 201 core targets with ischemic stroke. The core prevention targets of ischemic stroke include IL-6, AKT1, VEGFA, etc. The signaling pathways involved in core targets include AGE-RAGE signaling pathway, HIF-1 signaling pathway, and cAMP signaling pathways, etc. Then, the antiplatelet effect of phenolic acids was screened by *in vitro* antiplatelet experiment. Our results showed that phenolic acids have a good inhibitory effect on ADP-induced platelet aggregation and salvianolic acid A had a good antiplatelet effect. We further demonstrated that SAA preventive administration reduced neurobehavioral scores, decreased infarct size, and protected tight junction proteins in autologous thrombus stroke model. These studies not only shed light on the potential mechanisms of phenolic acids active components on ischemic stroke, but also provided theoretical and experimental information for the development of new medicines from Danshen for the prevention of ischemic stroke. In addition, our results suggest that SAA has the potential to be a candidate for ischemic stroke prevention drug.

## 1 Introduction

Ischemic stroke has a very high mortality and morbidity in the globe (Datta et al., 2020). It brings heavy economic burden to low- and middle-income countries (Saini et al., 2021). Primary prevention of ischemic stroke is of great significance. Preventive measures include lifestyle and diet modification, intervene with some risk factors including hypertension, antiplatelet therapy, and anticoagulation, etc. (Diener and Hankey, 2020). However, although ischemic stroke is a preventable and controllable disease, the prevention and control effect are not satisfactory (De Backer, 2017; Liu et al., 2021a). Therefore, the primary prevention of first stroke is a high priority.

At present, drugs commonly used to prevent ischemic stroke, including antiplatelet aggregators drugs (Hao et al., 2018), antihypertensive drugs (Brunström and Carlberg, 2018), antioxidant (Bahonar et al., 2017), anticoagulant (Beyer-Westendorf et al., 2017), lipid regulating agent (Khazaei et al., 2020), etc. However, although these drugs prevent ischemic stroke from different pharmacological effects, achieved limited effect in clinical application. In addition, some drugs have adverse reactions such as intracerebral hemorrhage. Aspirin as the classic antiplatelet drug, it is the commonly used in ischemic stroke prevention with increased bleeding events. There is no doubt about the effect of aspirin in clinical secondary stroke prevention (Florescu et al., 2019; Johnston et al., 2020). However, aspirin used in the primary prevention of ischemic stroke remains controversial (Xie et al., 2019). Harms outweighed the benefits with aspirin primary prevention (Bowman et al., 2018; McNeil et al., 2018). Moreover, other existing preventive drugs are also not effective in preventing ischemic stroke, such as clopidogrel, rosuvastatin, vitamin E, etc. Therefore, research and development of safe and effective drugs to prevent stroke may be one of the priorities of researchers.

Danshen is the dried root of Salvia miltiorrhiza Bge. It has a variety of pharmacological activities, which has been used for the treatment of cardiovascular many years. The ingredients of Danshen includes water-soluble components, for instance, phenolic acids, including salvianolic acids A (SAA), salvianolic acid B (SAB), C, etc., and lipid-soluble components, for instance, tanshinone I, II, isotanshinone, etc (Du et al., 2020). Nowadays, the protective pharmacological effects of lipid-soluble components in the pathogenesis of atherosclerosis, diabetes mellitus, and cancer were investigated (Guo et al., 2020). In addition, it has been reported the pharmacological effects of water-soluble components, and suggested that salvianolic acids had excellent pharmacological activity compared with other compounds in Danshen, especially SAA, SAB, etc. Previous research proved salvianolic acids had strong antioxidative (Huang and Zhang, 1992), anti-thrombic (Huang et al., 2010), inhibit platelet aggregation (Fan et al., 2010; Huang et al., 2010), and improve regional cerebral blood flow activities (Han et al., 2008). Meanwhile, Salvia Miltiorrhiza polyphenolic acid injection has a good therapeutic effect on cardiovascular and cerebrovascular diseases (Cong et al., 2022). Compared with lipid-soluble components, phenolic acids are better and more widely used in cardiovascular system. However, up to date, the screening process of phenolic acids in primary prevention of ischemic stroke has not been reported fully.

Network pharmacology seeks targets of drugs and diseases from public databases to analyze the mechanism of drug action from the perspective of biological balance, which helps to elucidate the interactions among compounds, genes, and diseases (Li et al., 2020; Wang et al., 2021a; Wang et al., 2022). In this paper, we first used the method of network pharmacology to predict the effect of phenolic acids in the prevention of ischemic stroke, and then screened the antiplatelet effect through *in vitro* antiplatelet experiment. At last, we further explored effect of the screened phenolic acids compounds-SAA on autologous thrombus stroke model. This study provided theoretical and experimental information for the development of new ischemic stroke preventive drug from phenolic acids.

## 2 Methods

### 2.1 Collection of phenolic acids and their targets

The main components of phenolic acids were found through literature review (Liang et al., 2016a), and the SMILE structure of the phenolic acids was obtained through PubChem database. Targets of phenolic acids were collected from the database of SwissTargetPrediction, STITCH, and CTD. Then we used the UniProt database to normalized the acquired targets. Gene ID of target obtained from Uniport database, species limited to ‘‘*Homo sapiens*’’. (The website of the public databases was shown in Table 1)

**TABLE 1:** Software and database for network pharmacology.

Name	Website
PubChem	https://pubchem.ncbi.nlm.nih.gov/
SwissTargetPrediction	http://www.swisstargetprediction.ch/index.php
STITCH	http://STITCH.embl.de/
CTD	http://ctdbase.org/
Uniprot	https://www.Uniprot.org/
GeneCards	https://www.genecards.org/
OMIM	https://www.OMIM.org/
Drugbank	https://www.drugbank.ca/
Venn diagram	http://bioinformatics.psb.ugent.be/webtools/Venn/
String	https://String-db.org/
PubChem	https://pubchem.ncbi.nlm.nih.gov/
David	https://david.ncifcrf.gov/
Metascape	https://www.metascape.org/

### 2.2 Identification of ischemic stroke-related targets

GeneCards, OMIM and Drugbank databases were accessed to collect ischemic stroke-related genes using the search terms “ischemic stroke” and “cerebral infarction”. After summarizing the three database targets, the duplicate targets were deleted. The overlapping targets of ischemic stroke targets and phenolic acids molecular targets, analyzed by Venn diagram online analysis tool were selected as candidate targets. (The website of the public databases and analysis tool was shown in Table 1).

### 2.3 Construction and analysis of the PPI network

The potential prevention targets of phenolic acids against ischemic stroke were the intersection of collected drug targets and ischemic stroke-related targets. The String database contains reported or predicted PPI relationships. The interactions between proteins were analyzed by String online tool (Szklarczyk et al., 2019). Cytoscape3.7.1 software was used to visualized the PPI network. Topological parameters in the network were calculated by the network analyzer plugin in Cytoscape3.7.1. (Table 1).

### 2.4 Construction of pathway analyses

KEGG and GO enrichment analysis were used to predicted the possible prevention targets of phenolic acids in the prevention of ischemic stroke. The enrichment information was analyzed by the Metascape online tool. *p* < 0.05 predicted by GO and KEGG were considered significantly enriched.

### 2.5 Antiplatelet aggregation assay of phenolic acids *in vitro*

The rats were anesthetized (intraperitoneal injection of chloral hydrate) and fixed to the rat plate. Blood was taken from the rat abdominal aorta and drawn into the siliconized vacutainers (3.8% sodium citrate). The methods of *in vitro* antiplatelet aggregation assay and the maximum aggregation rate of platelet were as reported in our previous literature (Wang et al., 2019). The platelet aggregation rate was determined at 37°C by using turbidimetric method with LG-PABER-I semiautomatic coagulation analyzer. The final concentration of inducer was as follows: ADP: 5 × 10^−6^ mol/L; AA: 5 × 10^−3^ mol/L; THR: 750 U/L.

### 2.6 Pharmacodynamic evaluation of ischemic stroke model

The ischemic stroke model by electrocoagulation and the methods of SAA preventive administration was established as previously described (Liu et al., 2021b). SAA (10 mg/kg, ig, purity >99% by HPLC, CAMS and PUMC, Beijing, China) and Aspirin (100 mg/kg, ig, Sigma, St. Louis, MO, United States) were preventive administered for 3 days, twice a day in the morning and evening, and the ischemic stroke model was prepared on the fourth day. After the rats were anesthetized with 4.5% isoflurane, the right common carotid artery (CCA), external carotid artery, and internal carotid artery (ICA) were bluntly separated and the external carotid artery was clamped with an artery clamp. The CCA was placed in the electric clamp (YLS-14B thrombus formation tester) and prestimulated for 1 min (1.00 mA) and then stimulated 4 min. Finally, the thrombus was crushed with homemade soft tweezers to block the ICA by itself and closed for another 15 min. After modeling, we immediately put the rats on an electric blanket to minimize pain and warmed with an electric lamp until they awoke.

After 24 h of ischemia, neurologic deficit score (0–18 score) was used to measure the degree of neurological impairment, the brain was harvested for TTC staining and cerebral hemorrhage detection. After taking blood from rats, the whole brain was carefully peeled off, frozen at −40°C quickly and sliced (2 mm). The slices were incubated with 0.5% TTC solution at 37°C for 15 min and then with 4% paraformaldehyde for 20 min. The slices were then placed in order and photographed. Image J was used to process and calculate the infarct size of each brain slice. The calculation method of cerebral infarction volume was as previously reported (Liu et al., 2021b).

After 24 h of ischemic stroke, the brain tissue was quickly removed after cardiac perfusion and frozen for 30 min before coronal section. After coronal sections were placed in order, cerebral hemorrhage score was performed, and the scoring standard was as follows: HI1 (small bleeding spot), HI2 (multiple fused spotty bleeds), PH1 (cerebral hematoma < 30% ischemic area), and PH2 (cerebral hematoma > 30% ischemic area). The brain tissue was homogenized with 0.01 mol·L^−1^ PBS at a ratio of 1∶1, centrifuge for 30 min at 12,000 ×g at 4°C. Brain hemoglobin detected by a Hemoglobin Assay Kit (BioAssay cat. DIHB −250).

### 2.7 Western blot assay

The method of total protein extraction and the protein concentration detected was the same as previously described (Liu et al., 2021b). The denatured sample was separated by SDS-PAGE, and then transferred onto a PVDF membrane. After transmembrane, 5% bovine serum albumin (BSA) was used to blocked the membrane for 2 h at room temperature. Then the primary antibodies (Table 2), including Occludin, Claudin-5, ZO-1, β-Actin were treated with the membranes at 4°C overnight. After incubation of primary antibody, TBST was used to wash away excess primary antibody 30 min at room temperature. Secondary antibodies (Table 2) against HRP-conjugated rabbit or mouse IgG were added for 2 h at room temperature. ImageJ software was used to calculated density of the band.

**TABLE 2:** Antibodies and the species.

Primary antibody	Specify source	Catalog#	Company
Anti-Occludin	Rabbit	ab216327	Abcam
Anti-Claudin-5	Rabbit	YT0953	Immunoway
ZO-1	Rabbit	21773-1-AP	Proteintech
β-Actin	Mouse	8H10D10	Cell signaling technology
HRP-conjugated rabbit IgG	Rabbit	CW0103S	CWBIO
HRP-conjugated mouse IgG	Mouse	CW0102S	CWBIO

### 2.8 Statistical analysis

All data were imported to GraphPad Prism 7.00 software for statistical analysis, and specified *p* < 0.05 has significant difference between groups. The comparison between multiple groups we applied one-way ANOVA followed by Dunnett’s multiple comparison test. The analysis between the two groups was performed by Unpaired Student’s t-test. Our experimental data were presented as the mean ± SEM.

## 3 Result

### 3.1 The candidate targets of phenolic acids against ischemic stroke

**FIGURE 1:**
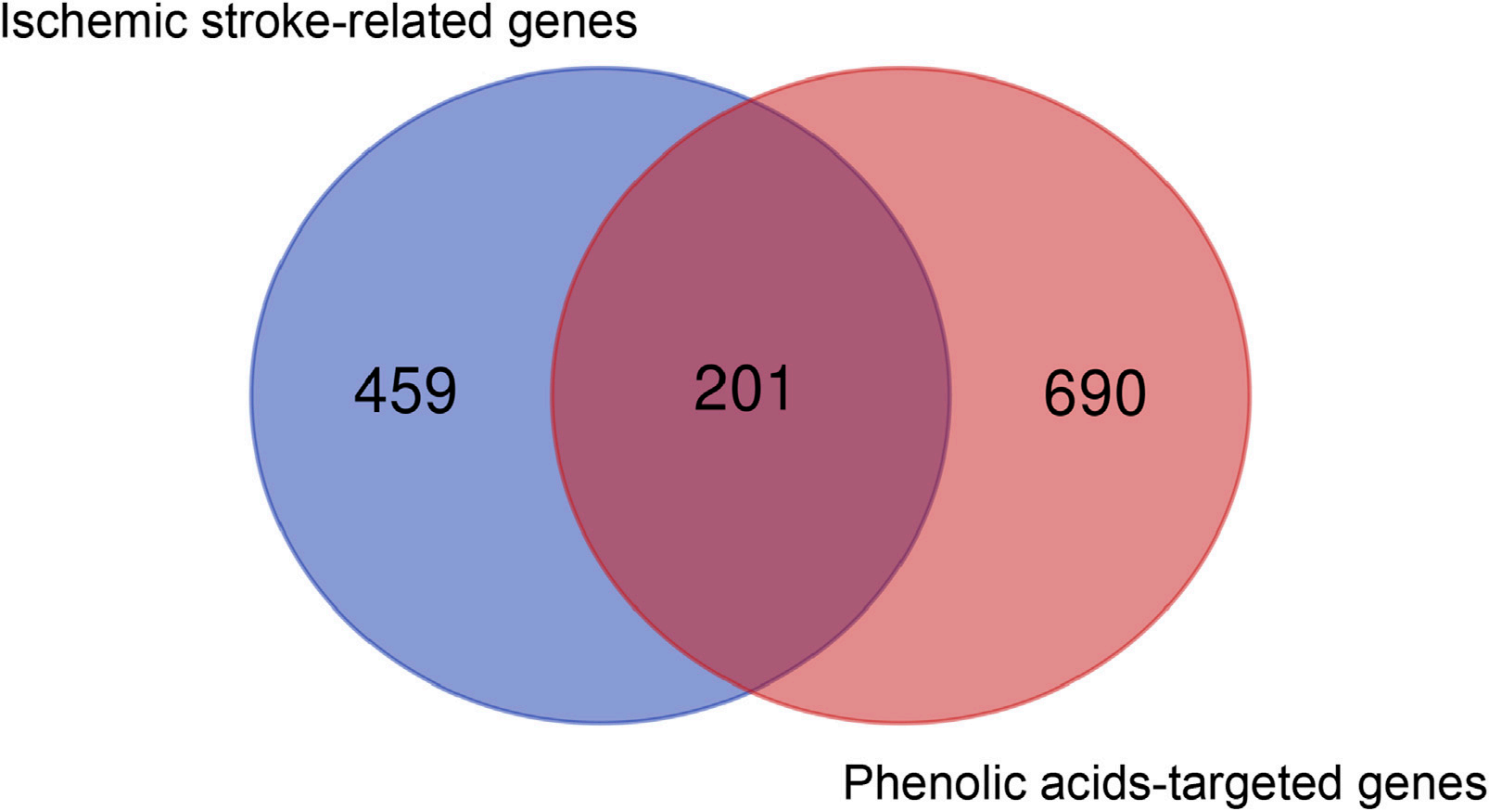
Analysis of 201 core genes between 660 ischemic stroke-associated genes and 891 phenolic acids—associated genes *via* Draw Venn Diagram.

A total of 39 main components of salvianolic acids were found through literature review (Liang et al., 2016a), as shown in Table 3. The SMILEs structures of the above 39 main components were obtained from PubChem database. Among of them, 18 SMILEs structure could not be found, so we hand-drawn the structures and predicted on SwissTargetPrediction website. However, 3-(3, 4-dihydroxyphenyl) lactamide, 1- hydroxy-pinoresinol-1-O-beta-D-glucoside, magnesium lithospermate B, and ammonium potassium lithospermate B components could not be predicted. Then 35 kinds of phenolic acids were predicted. The target genes related to the candidate compounds were screened by CTD, STITCH and SwissTargetPrediction databases. The Gene names and gene IDs were obtained from Uniprot database. Finally, a total of 891 soluble phenolic acids target genes were obtained (Figure 1).

**TABLE 3:** Phenolic acids of salvia miltiorrhiza.

No.	Name	Chemical structure
1	Danshensu	C_9_H_10_O_5_
2	Caffeic acid	C_9_H_8_O_4_
3	Salvianolic acid D	C_20_H_18_O_10_
4	Salvianolic acid E	C_36_H_30_O_16_
5	Ferulic acid	C_10_H_10_O_4_
6	Isoferulic acid	C_10_H_10_O_4_
7	Protocatechuic aldehyde	C_7_H_6_O_3_
8	Protocatechuic acid	C_7_H_6_O_4_
9	Ethyl lithospermate	C_29_H_26_O_12_
10	Salvianic acid C	C_18_H_18_O_9_
11	Salvianolic acid A	C_26_H_22_O_10_
12	Salvianolic acid B	C_36_H_30_O_16_
13	Salvianolic acid C	C_26_H_20_O_10_
14	Salvianolic acid F	C_17_H_14_O_6_
15	Salvianolic acid G	C_18_H_12_O_7_
16	Salvianolic acid I	C_27_H_22_O_12_
17	Salvianolic acid J	C_27_H_22_O_12_
18	Salvianolic acid L	C_37_H_34_O_16_
19	Salvianolic acid T	C_27_H_22_O_12_
20	Salvianolic acid U	C_27_H_22_O_12_
21	Rosmarinic acid	C_18_H_16_O_8_
22	Methyl rosmarinate	C_19_H_18_O_8_
23	Prolithospermic acid/przewalskinic acid	C_18_H_14_O_8_
24	Lithospermic acid	C_27_H_22_O_12_
25	Salviaflaside	C_24_H_26_O_13_
26	Salvinal	C_20_H_20_O_6_
27	3-(3,4-dihydroxyphenyl) lactamide	C_9_H_11_NO_4_
28	1-hydroxy-pinoresinol-1-o-β-D-glucoside	C_26_H_32_O_12_
29	Lithospermic acid monomethyl ester	C_28_H_24_O_12_
30	Lithopermic acid dimethyl ester	C_29_H_26_O_12_
31	Methyl salvianolic acid C	C_27_H_22_O_10_
32	Dimethyl lithospermate	C_29_H_24_O_12_
33	9″-methyl lithospermate	C_28_H_24_O_12_
34	Isosalvianolic acid C	C_26_H_20_O_9_
35	Ethyl lithospermate B	C_38_H_34_O_16_
36	Magnesium lithospermate B	C_36_H_28_MgO_16_
37	Ammonium-postassium lithospermate B	C_36_H_28_O_16_^2-^
38	2-(3-methoxy-4-hydroxyphenyl)-5-(3-hydroxypropyl)-7-methoxybenzofu-ran-3-caraldehyde	C_20_H_20_O_6_
39	Ailanthoidol	C_19_H_18_O_5_

We collected 660 genes associated with ischemic stroke from the GeneCards, OMIM and Drugbank databases. Ischemic stroke-related genes are mainly related to Ca2^+^ transport (67 related genes including MMP9, MMP3 and MPO, etc.), cell junction (40 related genes including KDR, OCLN and ITGB, etc.), blood coagulation (23 related genes including VWF, ADAMTS13 and F2, etc.), angiogenesis (20 related genes including MMP2, VEGFA, and NOTCH1, etc.), cytokines (24 related genes including IL-6, IL-1β, and TNF, etc.), immune response (15 related genes including HMGB1, IL-1β, and TLR4, etc.), oxidative reaction (8 related genes including MPO, GAPDH, and APP etc.), etc. These targets were associated with the pathological changes in stroke pathology. Through the online analysis tool Venn diagram, the predicted targets of phenolic acids and ischemic stroke were analyzed, then the overlapping targets obtained were used as the core targets of phenolic acids in the prevention of ischemic stroke. As shown in Figure 1, there were 201 intersecting genes between phenolic acids and ischemic stroke.

### 3.2 Construction and analysis of protein interaction network of phenolic acids for prevention of ischemic stroke

**FIGURE 2:**
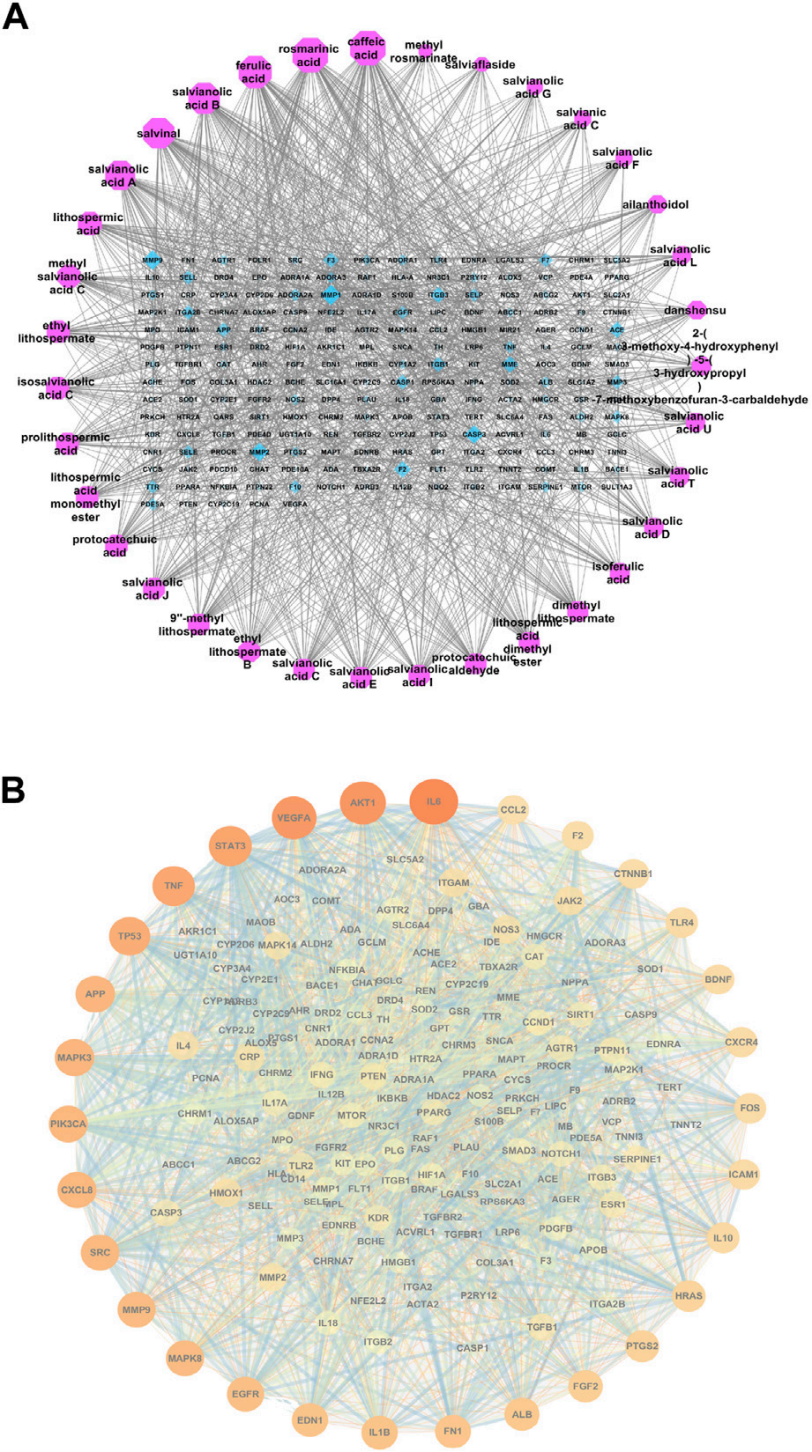
Phenolic acids—core target network **(A)** and top 30 targets in PPI network **(B)**.

We used Cytoscape 3.7.1 to construct 35 compounds and 201 target genes protein interaction visualized networks. As shown in Figures 2A, 6 of the 35 compounds were associated with ischemic stroke genes >50, including caffeic acid, rosmarinic acid, ferulic acid, SAB, salvinal, and SAA. Additionally, PPI network map of core targets was made by Cytoscape3.7.1, and the top 30 targets were selected according to Degree value. IL-6, AKT1, VEGFA, STAT3, TNFα, etc., were the core target of phenolic acids in preventing ischemic stroke (Figure 2B).

### 3.3 GO and KEGG enrichment analysis of potential target genes

**FIGURE 3:**
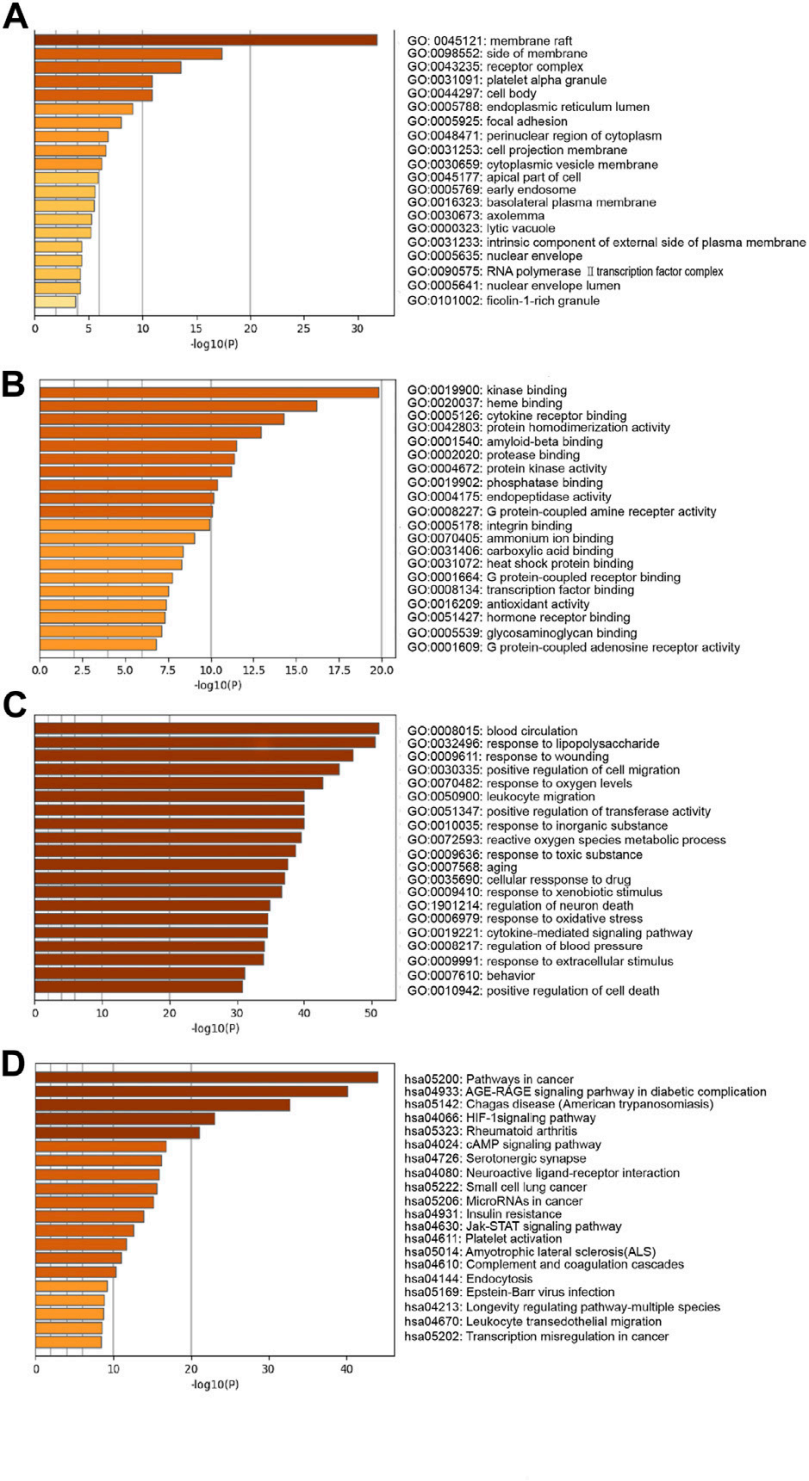
KEGG and GO enrichment analysis. GO enrichment diagram of phenolic acids and cerebral ischemia hub target. **(A)** “cellular components”; **(B)** “molecular function”; **(C)** “biological process”. Sort by −Log10 (*p*-value) importance of each pathway. **(D)** KEGG pathway analysis of phenolic acids and ischemic stroke core target. Sort by −Log10 (*p*-value) importance of each pathway.

201 potential targets analyzed by GO and KEGG pathway enrichment performed the underlying mechanism of phenolic acids in the prevention of ischemic stroke. As shown in Figures 3A–C, GO analysis results predicted the effect of SAA from cellular component (CC), molecular function (MF) and biological process (BP) aspects. The results of CC analysis revealed that there was more protein in the membrane raft. The MFs were mainly associated with “kinase binding”, “heme binding”, “cytokine receptor binding”, “protein homodimerization activity”, etc. The main BPs were “blood circulation”, “response to lipopolysaccharide”, “response to wounding”, etc.

Furthermore, we predicted the signaling pathways of these target genes by KEGG pathway analysis. These signaling pathways were ranked according to the degree of enrichment (*p* < 0.05), and the top 6 was cancer pathway, AGE-RAGE signaling pathways, chagas disease, HIF-1 signaling pathway, rheumatoid arthritis, and cAMP signaling pathways. Among these signaling pathways, AGE-RAGE, HIF-1, and cAMP signaling pathways were closely related to ischemic stroke pathophysiological process (Figure 3D).

### 3.4 The *in vitro* antiplatelet effect of phenolic acids

**FIGURE 4:**
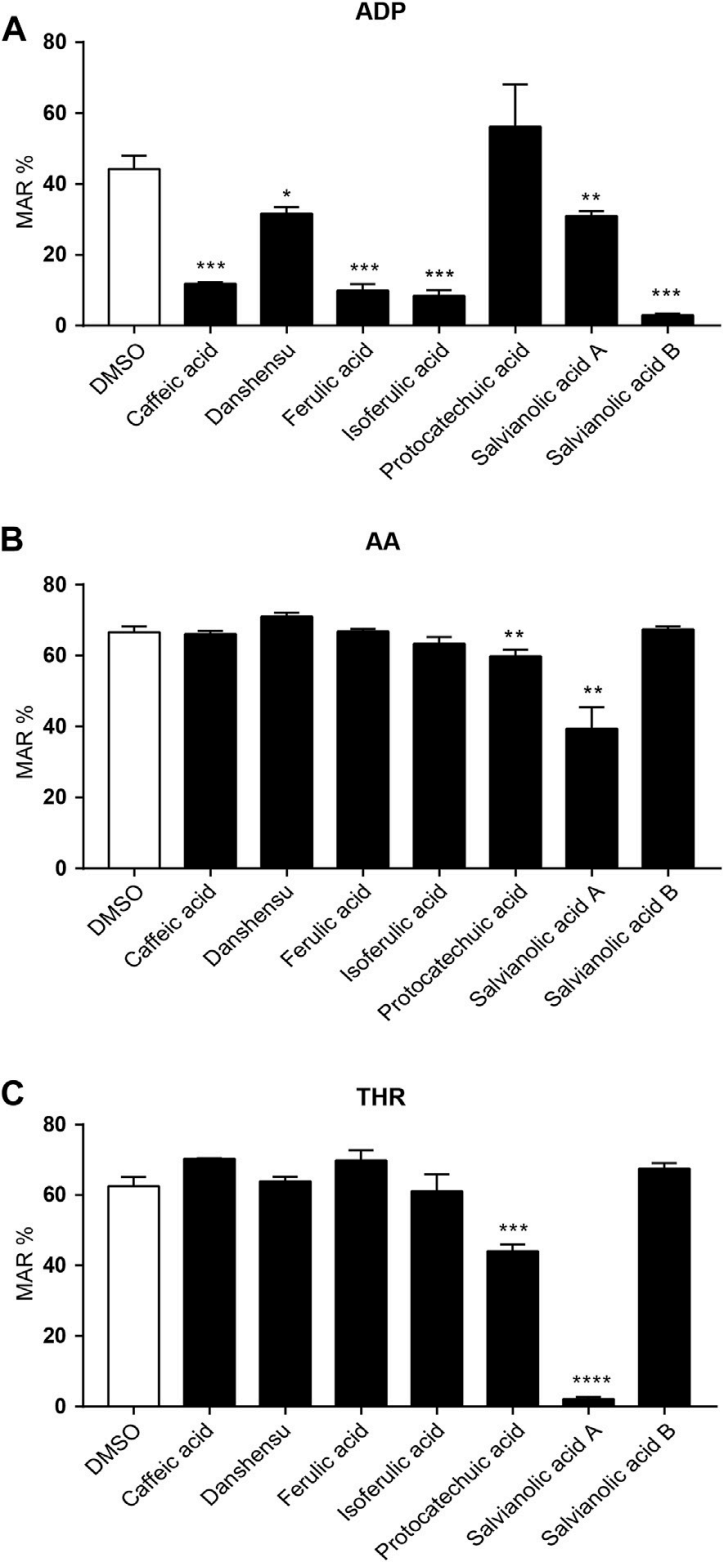
Effect of phenolic acids on ADP, AA, THR-induced platelet aggregation *in vitro*. Platelet aggregation was induced by adenosine diphosphate (ADP: 5 × 10^–6^ mol/L, **(A)**, arachidonic acid (AA: 5 × 10^–3^ mol/L, **(B)** and thrombin (THR:750 U/L, **(C)**, respectively. *n* = 3, mean ± SEM. *p < 0.05, **p < 0.01, ***p < 0.001 and ****p < 0.0001 vs. DMSO group.

We selected several phenolic acids to prevent ischemic stroke screened by network pharmacology and carried out *in vitro* antiplatelet experiments. Our results demonstrated that caffeic acid, danshensu, ferulic acid, isoferulic acid, SAB and SAA effectively inhibited ADP induced platelet aggregation (Figure 4A). As shown in Figure 4B, protocatechuic acid and SAA obviously inhibited AA and THR induced platelet aggregation. Therefore, combined with the *in vitro* antiplatelet results, phenolic acids exerted a better inhibitory effect on platelet aggregation induced by ADP and SAA played a good inhibitory effect on platelet aggregation induced by the different platelet inhibitors *via* analyzed comprehensively.

### 3.5 Preventive effects of SAA in autologous thrombus stroke rats

**FIGURE 5:**
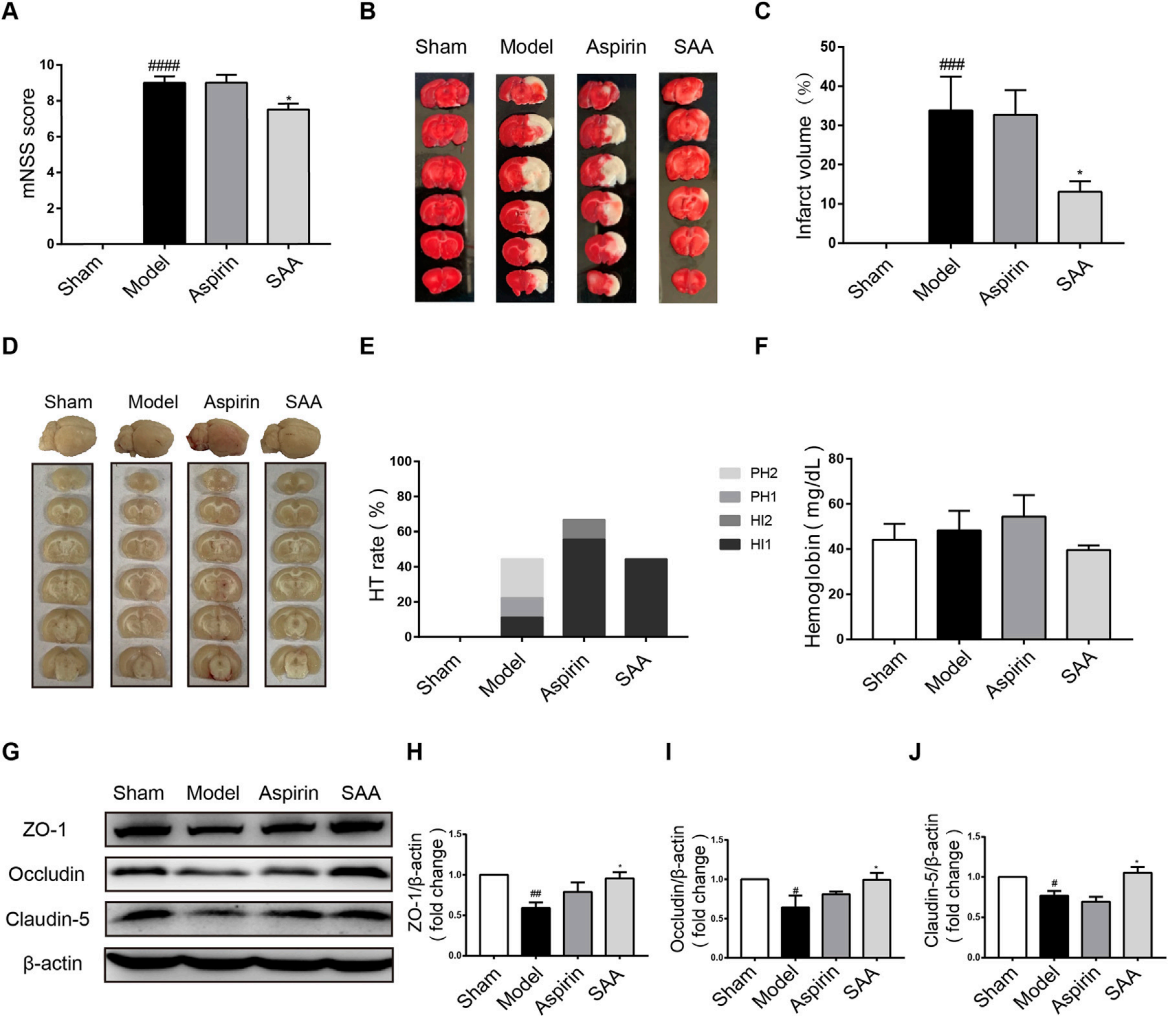
Preventive effects of SAA on rat ischemia injury. **(A)** mNSS at 24 h after ischemia with preventive administration of SAA. **(B, C)** The effects of SAA on cerebral infarction with 3d preventive administration. **(D)** The occurrence of hemorrhagic transformation in brain after SAA preventive administration. **(E)** Incidence of HT after SAA preventive administration. **(F)** The content of cerebral hemoglobin after SAA preventive administration. **(G–J)** Representative Western blot of Occludin, Claudin-5 and ZO-1 after SAA preventive administration 3d (*n* = 4). *n* = 6, mean ± SEM, #*p* < 0.05, ##*p* < 0.01, ###*p* < 0.001, and #### *p* < 0.0001 vs sham group, *p < 0.05 vs model group.

We then tested the efficacy of preventive administration of SAA in autologous thrombus stroke model by electrocoagulation to further prove the preventive effect of SAA on ischemic stroke. Compared with the model group, preventive administration of SAA obviously reduced the mNSS and cerebral infarct volume of the ischemic rats (Figures 5A–C). Our results demonstrated that SAA have significantly improved effect on neurological deficits and brain edema in ischemic stroke rats.

24 h after ischemic stroke, the hemorrhage transformation (HT) occurred in ischemic model, aspirin, and SAA group. The results of brain slices showed that there was a slight hemorrhage in the model and aspirin group, while SAA preventive administration group could not significantly decrease the level of hemorrhage (Figures 5D–F). Therefore, SAA had no protect effect on HT caused in ischemic stroke rats with 3d preventive administration.

TJ proteins contribute to BBB integrity. Compared with the sham group, ZO-1, occludin and claudin-5 was decreased in the model group detected by Western Blot. While, SAA reversed ZO-1, occludin and claudin-5 protein expression (Figures 5G–J). Therefore, SAA prevented TJ degradation in ischemic stroke rats.

## 4 Discussion

Phenolic acids exert various pharmacological effects on the cardiovascular system, such as protect against cerebral ischemia injury, antiplatelet aggregation, antioxidation, improving the disorders in memory functions, improving microcirculation, and promoting tissue repair, etc (Du and Zhang, 1997; Chen et al., 2000; Fan et al., 2010; Hou et al., 2016; Li et al., 2017), which were widely used in clinic for the treatment of stroke, nephropathy, etc. Salvia Miltiorrhiza polyphenolic acid injection has been used in clinical practice with the indication of blood stasis in recovery period of mild to moderate cerebral infarction, with the effects of improve cognitive, motor functions, and promote neurological recovery (Cong et al., 2022). Therefore, phenolic acids in Salvia Miltiorrhiza had an unquestionable effect in the treatment of ischemic stroke. However, which component of these phenolic acids is better in primary prevention of ischemic stroke has not been studied. Considering the interconnectedness of genes for prevention and treatment of ischemic stroke, we aimed to explore preventive effect of phenolic acids on ischemic stroke through network pharmacology analyses.

**FIGURE 6:**
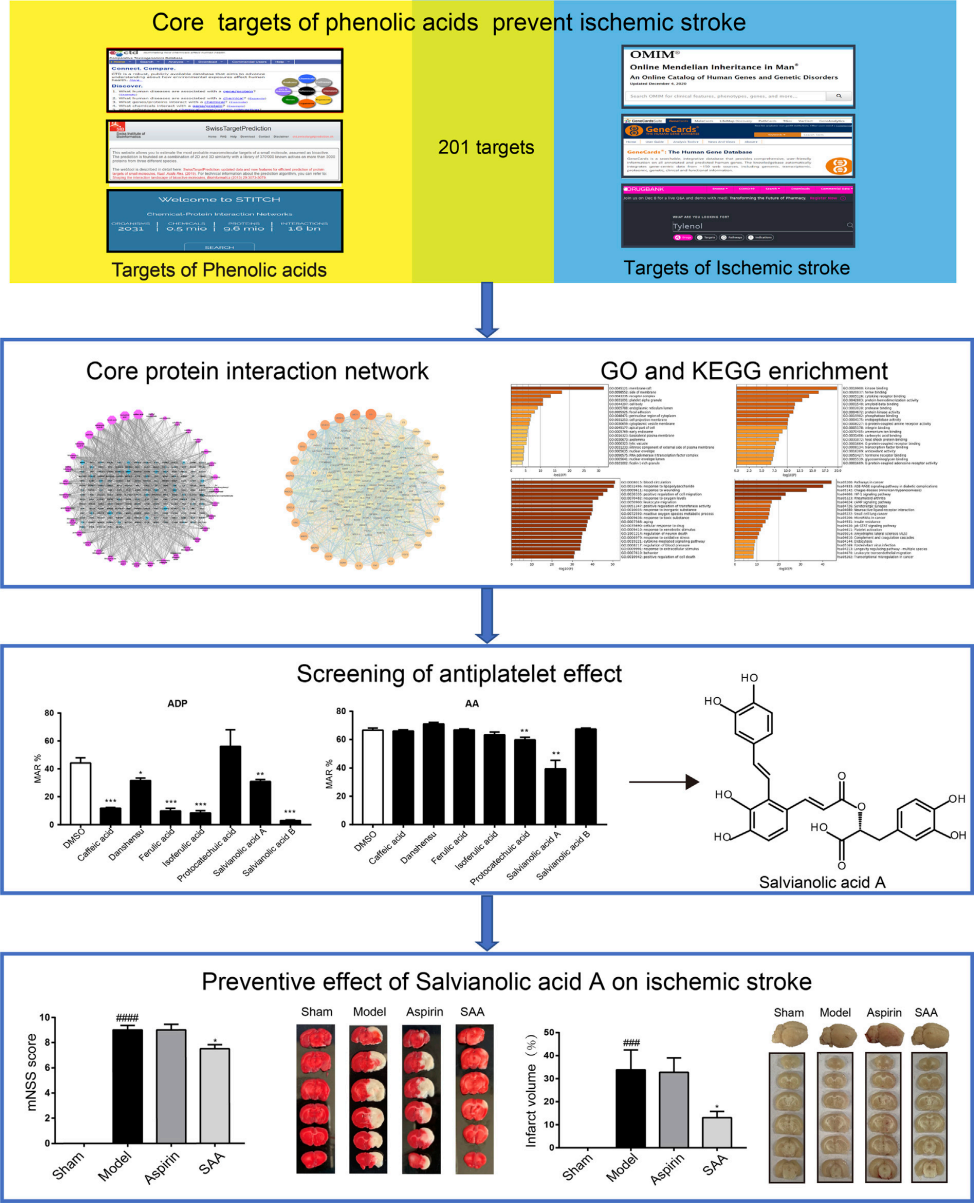
Work scheme of the present study.

In this study, we screened the preventive effect of phenolic acids on ischemic stroke and investigate its mechanism by network pharmacology. The network pharmacological analysis ofphenolic acids identified 6 monomeric compound (>50 genes, such as caffeic acid, rosmarinic acid, ferulic acid, salvianolic acid B,salvinal, salvianolic acid A), 30 genes (IL-6, AKT1, VEGFA, STAT3, TNF, TP53, APP, etc.), and 20 target gene-regulated pathways (AGE-RAGE, HIF-1, cAMP signaling pathway, etc.) associated with ischemic stroke. In addition, our results demonstrated that SAA has a good antiplatelet effect screened by *in vitro* antiplatelet assay. Moreover, in an autologous thrombus stroke model, we proved that preventive administration of SAA had neuroprotective effects, reduced infarct volume, and protected tight junction proteins after ischemic stroke (Figure 6). Therefore, our experiments demonstrated that phenolic acids had the effect on preventing stroke and SAA had the potential to be a preventive drug for ischemic stroke.

Combined network pharmacology and existing literature, we think the ideal drugs to prevent ischemic stroke should have the following characteristics (Du et al., 2018; Liu et al., 2021a): 1) regulate blood status, including blood coagulation or platelet overactivation. 2) protect cerebrovascular, including protect BBB integrity, prevent endothelial damage and inhibit inflammation etc. 3) manage risk factors, such as blood pressure, diabetes, hyperhomocysteinemia, etc. Therefore, the ideal stroke prevention drugs should have a multiple regulatory effect on the blood, blood vessels and risk factors, to play a good preventive role.

Network pharmacology results demonstrated that caffeic acid, rosmarinic acid, ferulic acid, SAB, salvinal, and SAA, etc., had good regulatory effect on the core targets of ischemic stroke prevention. It has been reported that these screened salvia miltiorrhiza compounds have protective effects on cerebral ischemia (Liang et al., 2015; Wang et al., 2016; Ren et al., 2017; Zhang et al., 2018; Wang et al., 2021b). Caffeic acid (Lu et al., 2015), rosmarinic acid (Zou et al., 1993), ferulic acid (Choi et al., 2018), SAB (Zheng et al., 2021), SAA (Fan et al., 2010) has antiplatelet and antithrombotic effects. It is reported that caffeic acid (Taïlé et al., 2022), rosmarinic acid (Luan et al., 2013), ferulic acid (Salau et al., 2020), SAB (Li et al., 2010), and SAA (Liu et al., 2021b) protects BBB integrity. Moreover, caffeic acid (Khan et al., 2007), rosmarinic acid (Luan et al., 2013), ferulic acid (Koh, 2013), SAB (Lv et al., 2015), SAA (Ling et al., 2021) alleviated inflammatory response caused by ischemic stroke. However, the existing studies were most about the therapeutic effect of phenolic acids on ischemic stroke, and few studies were about the preventive effect of phenolic acids on ischemic stroke. Based on the similarity of targets for the treatment and prevention of ischemic stroke, and existing experiments had proved that phenolic acids meet the conditions of ideal preventive drugs for stroke prevention, we preliminary hypothesized that phenolic acids screened by network pharmacology may have a good effect on preventing ischemic stroke.

In order to pinpoint the key targets of phenolic acids for stroke prevention, we analyzed the core targets by gene-phenotype correlation analyses. Our results demonstrated that IL-6, AKT1, VEGFA, STAT3, TNF, TP53, APP, etc, genes are significantly related to the prevention of ischemic stroke. IL-6 and TNF are pleiotropic cytokine with significant functions in the immune system (Lambertsen et al., 2012; Yao et al., 2014). Inflammation is a hallmark of stroke pathology, and the cytokines is related with infarct evolution (Lambertsen et al., 2012). Akt-related pathways is associated with cell migration, proliferation, apoptosis, oxidative stress, etc., during stroke (Gu et al., 2022). VEGFs participated in the process of cerebral edema, neuroprotection, angiogenesis, etc., in ischemic stroke rats (Greenberg and Jin, 2013). STATs are involved in innate immunity, cell death, organogenesis, etc (Liang et al., 2016b). It is reported that salvianolic acids promoted angiogenesis and neurological recovery by regulating STAT3 signaling pathway after ischemia (Li et al., 2017). In addition, Wang et al. proved that the core target of ischemic stroke in Fufang Xueshuantong treatment was STAT1, STAT3 through the method of network pharmacology, which was consistent with our predicted results (Wang et al., 2022). Therefore, these core genes inhibit inflammation and apoptosis, protect vascular endothelial injury in order to inhibit thrombosis in ischemic stroke rats.

GO and KEGG analysis results further demonstrated several BP and signaling pathways in the prevention of ischemic stroke by phenolic acids. The enriched results of BP including “blood circulation”, “response to lipopolysaccharide”, “response to wounding”, and “positive regulation of cell migration”, etc. The enriched KEGG pathways including AGE-RAGE, HIF-1, and cAMP signaling pathways, etc. By analyzing the results of GO and KEGG enrichment, we found that these processes were closely related to key genes associated with cerebral ischemia. Inhibition of AGE-RAGE signaling pathway could inhibit neuroinflammation and cell apoptosis caused by ischemic stroke (Liu et al., 2021c; Wang et al., 2021c). The increase of circulating soluble RAGE (sRAGE) after ischemic and hemorrhagic stroke may be a candidate biomarkers related to prognostic or follow-up value (Menini et al., 2014). During stroke, HIF-1 signaling pathway was an important regulator, which was related to the process of angiogenesis, cell survival, glucose metabolism, etc. (Pan et al., 2021). The underlying mechanisms of HIF-1 regulated stroke including apoptotic (Cheng et al., 2014), neurogenesis (Wu et al., 2018), BBB integrity (Liu et al., 2021b) inflammation (Pan et al., 2021), autophagy (Xie et al., 2022; Zhang et al., 2022), and oxidative stress (Peng et al., 2020). However, the effect of HIF-1 was double-sided and may be related to the time and degree of ischemia. cAMP signaling pathways was related with the process of neuronal survival (Martin et al., 2005; Kitagawa, 2007), motor functional recovery and axonal regeneration (Gao et al., 2020), apoptosis (Zhang et al., 2020). To sum up, AGE-RAGE, HIF-1, and cAMP signaling pathways were the key regulating agent in the development and prevention of ischemic stroke.

In our previous study, we had demonstrated that the antiplatelet effect of SAA was mild in a thrombus model (Wang et al., 2019). Based on the previous experimental approaches, we studied the preventive effect of SAA in the autologous thrombus stroke model with 3d preventive administration. Our results proved that preventive administration of SAA reduced neurobehavioral scores, infarct size, and had a better protective effect on tight junction proteins, compared with model group. However, SAA had no effect on intracerebral hemorrhage. It is important to maintain the integrity of BBB to protect cerebral vessels in order to prevent thrombosis and cerebral hemorrhage. Meanwhile, the core targets predicted by network pharmacology, such as AKT1, VEGFA, STAT3 have regulatory effects on BBB. Our results demonstrated that SAA had a protective effect on marker protein of BBB, including ZO-1, Occludin, Claudin-5. However, SAA has no effect on cerebral hemorrhage, which may be related to the duration of preventive administration. Appropriate extension of the duration of preventive administration may enhance its protective effect. After years of research, we found that SAA had an extensive pharmacological activity, such as antiplatelet, anti-thrombus, anti-inflammatory, and antioxidative, etc. It has been reported that SAA has more extensive effects than other salvianolic acids in Danshen (Du et al., 2020). Our previous experiments have preliminary proved that SAA prevented ischemic stroke by protecting cerebrovascular endothelial injury (Liu et al., 2021b). Therefore, SAA has a good preventive effect on acute ischemic stroke, and its effect and mechanism need further experimental proof.

The pathogenesis of ischemic stroke is very complex, so it is difficult to prevent stroke. At present, the prevention and control strategies for ischemic stroke are not mature, and the effect of preventive drugs are not ideal. Therefore, finding the targets of ischemic stroke prevention is of great significance. In this article, we predicted the targets of ischemic stroke prevention by network pharmacology method. Our results proved that the targets of ischemic stroke prevention were associated with blood, blood vessels and other risk factors. These results suggested that the prevention of ischemic stroke should be carried out with multiple targets and multiple directions. The SAA we screened from network pharmacology not only has inhibitory effects on platelets, but also has protective effects on vascular endothelial cells. It has the potential to develop into a preventive drug for ischemic stroke.

In conclusion, network pharmacological predicted that phenolic acids including caffeic acid, rosmarinic acid, ferulic acid, SAB, and SAA, etc., had a good preventive effect on ischemic stroke. The core prevention targets and pathways include IL-6, AKT1, VEGFA targets, and AGE-RAGE, HIF-1, cAMP signaling pathways, etc. Moreover, SAA may be a preventive drug candidate of ischemic stroke screened by experiment.

## Data Availability

The original contributions presented in the study are included in the article/Supplementary Material, further inquiries can be directed to the corresponding authors.
