# Corrigendum: Network pharmacology and experimental study of phenolic acids in salvia miltiorrhiza bung in preventing ischemic stroke

**DOI:** 10.3389/fphar.2023.1155574

**Published:** 2023-02-13

**Authors:** Chengdi Liu, Lida Du, Sen Zhang, Haigang Wang, Linglei Kong, Guanhua Du

**Affiliations:** ^1^ Department of Pharmacy, Affiliated Beijing Friendship Hospital, Capital Medical University, Beijing, China; ^2^ Beijing Key Laboratory of Drug Targets Identification and Drug Screening, Institute of Materia Medica, Chinese Academy of Medical Sciences and Peking Union Medical College, Beijing, China; ^3^ Department of Surgery, University of Toronto, Toronto, ON, Canada

**Keywords:** phenolic acids, salvianolic acid A, ischemic stroke, network pharmacology, prevention drug, antiplatelet experiment

In the published article, there was an error in Figure 6 as published. Figure 6 is missing in the published article**.** The corrected Figure 6 and its caption appear below.

**FIGURE 6:**
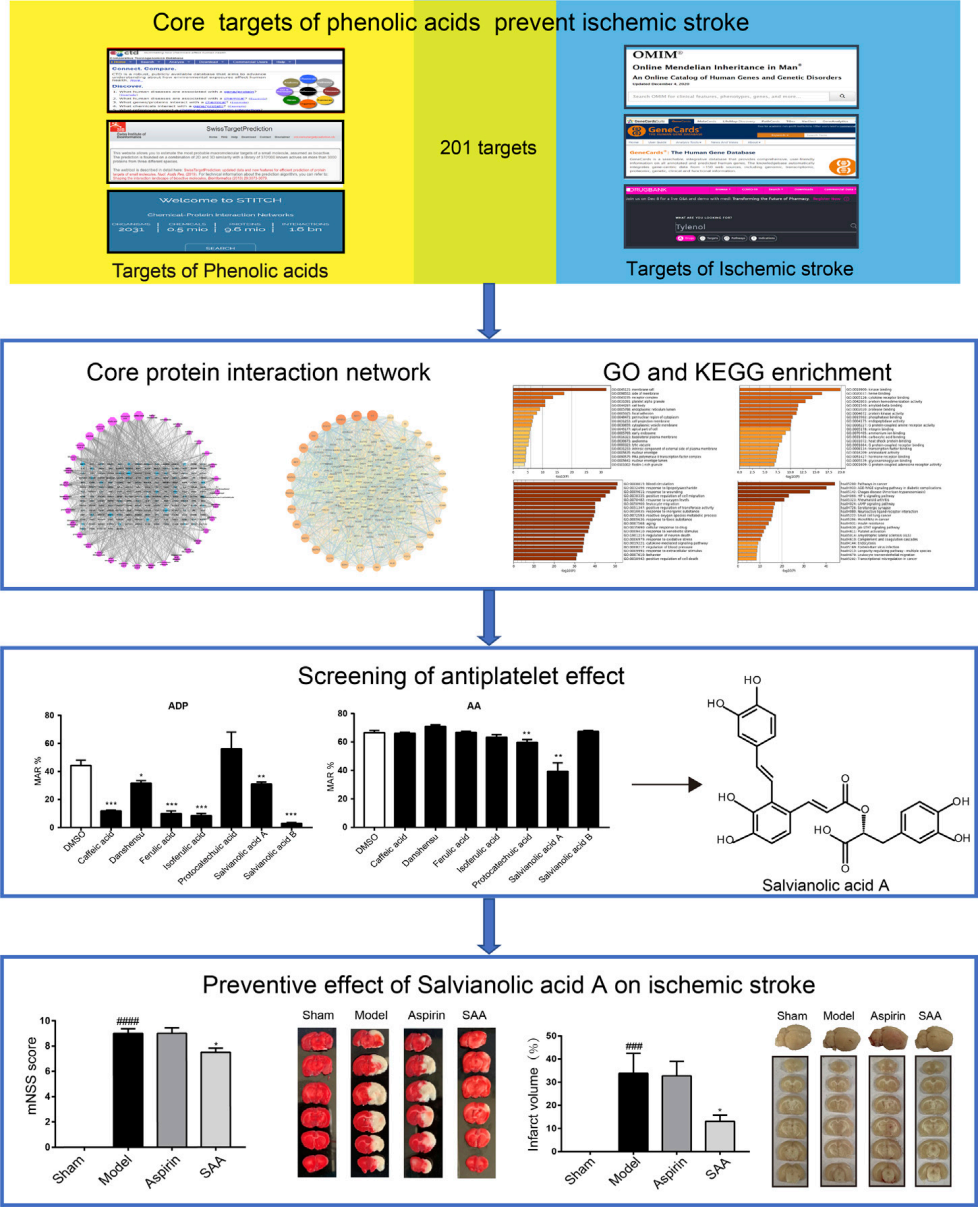
Work scheme of the present study.

The authors apologize for this error and state that this does not change the scientific conclusions of the article in any way. The original article has been updated.

